# MEK inhibition leads to BRCA2 downregulation and sensitization to DNA damaging agents in pancreas and ovarian cancer models

**DOI:** 10.18632/oncotarget.24294

**Published:** 2018-01-22

**Authors:** Francesca Vena, Ruochen Jia, Arman Esfandiari, Juan J. Garcia-Gomez, Manuel Rodriguez-Justo, Jianguo Ma, Sakeena Syed, Lindsey Crowley, Brian Elenbaas, Samantha Goodstal, John A. Hartley, Daniel Hochhauser

**Affiliations:** ^1^ Cancer Research UK Drug-DNA Interactions Research Group, UCL Cancer Institute, Paul O’Gorman Building, University College London, London WC1E 6DD, UK; ^2^ Department of Research Pathology, UCL Cancer Institute, London EC1M6BQ, UK; ^3^ EMD Serono Research and Development Institute, Billerica 01821, MA, USA

**Keywords:** DNA damage, pancreatic cancer, ovarian cancer, targeted therapy, MEK inhibitors

## Abstract

Targeting the DNA damage response (DDR) in tumors with defective DNA repair is a clinically successful strategy. The RAS/RAF/MEK/ERK signalling pathway is frequently deregulated in human cancers. In this study, we explored the effects of MEK inhibition on the homologous recombination pathway and explored the potential for combination therapy of MEK inhibitors with DDR inhibitors and a hypoxia-activated prodrug.

We studied effects of combining pimasertib, a selective allosteric inhibitor of MEK1/2, with olaparib, a small molecule inhibitor of poly (adenosine diphosphate [ADP]-ribose) polymerases (PARP), and with the hypoxia-activated prodrug evofosfamide in ovarian and pancreatic cancer cell lines. Apoptosis was assessed by Caspase 3/7 assay and protein expression was detected by immunoblotting. DNA damage response was monitored with γH2AX and RAD51 immunofluorescence staining. *In vivo* antitumor activity of pimasertib with evofosfamide were assessed in pancreatic cancer xenografts.

We found that BRCA2 protein expression was downregulated following pimasertib treatment under hypoxic conditions. This translated into reduced homologous recombination repair demonstrated by levels of RAD51 foci. MEK inhibition was sufficient to induce formation of γH2AX foci, suggesting that inhibition of this pathway would impair DNA repair. When combined with olaparib or evofosfamide, pimasertib treatment enhanced DNA damage and increased apoptosis. The combination of pimasertib with evofosfamide demonstrated increased anti-tumor activity in BRCA wild-type Mia-PaCa-2 xenograft model, but not in the BRCA mutated BxPC3 model.

Our data suggest that targeted MEK inhibition leads to impaired homologous recombination DNA damage repair and increased PARP inhibition sensitivity in BRCA-2 proficient cancers.

## INTRODUCTION

The Ras/Raf/MEK/ERK signalling pathway regulates key cellular processes such as proliferation, differentiation and survival [1]. Targeted therapies that inhibit components of this pathway have been developed for the treatment of cancers where this signalling cascade is aberrantly activated [2, 3]. An example is the successful experience with inhibitors of the MAPK pathway in melanoma, where highly selective inhibitors of BRAF and MEK1/2 kinases have shown antitumor responses in both preclinical settings and have been approved for use in BRAF or NRAS mutant melanoma [4].

Breast cancer susceptibility genes 1 and 2 (BRCA1/2) are required for DNA double strand break repair mediated by homologous recombination (HR). Deficiency in BRCA1 or BRCA2 is associated with the inability of repair DNA breaks, thus leading to chromosomal instability [5]. Germline mutations in either BRCA1 or BRCA2 are associated with increased predisposition to breast, ovarian and other cancers [6]. PARP inhibitors have shown promising antitumor activity in patients with impaired HR repair [7] and have been recently approved by the FDA for treatment of ovarian cancer patients carrying BRCA1 and BRCA2 mutations. PARP inhibitors act by blocking the enzymatic activity of PARP protein (Poly ADP-ribose polymerase), which is involved in the repair of DNA strand breaks. This is the basis of selective activity of PARP inhibitors in BRCA-deficient tumors [8], It would be significant if tumors expressing BRCA1/2 wild type but with “BRCAness” phenotype [9] could be sensitized to DNA damaging agents, thus expanding the potential use of these compounds in a wider patient population.

In the present study, we determined that the selective allosteric MEK1/2 inhibitor pimasertib (AS703026/MSC1936369) increased DNA damage by downregulating BRCA2, thus enhancing activity of PARP inhibitors in BRCA2 proficient cell lines. We showed enhanced antitumor activity of evofosfamide (TH-302), a hypoxia-activated pro-drug selectively activated under low oxygen conditions to release the DNA cross-linker bromo-isophosphoramide mustard, in combination with pimasertib in human pancreatic cancer cell lines, an effect modulated through altered BRCA2 expression.

## RESULTS

### Pimasertib sensitizes pancreatic and ovarian cancer cells to olaparib treatment by reducing BRCA2 protein expression

The tumor microenvironment is characterized by hypoxia, which contributes to cancer cell growth and dissemination [10]. In addition, hypoxia causes aberrant expression of genes involved in DNA repair mechanisms [11]. In this study, we investigated the effects of MEK targeted therapy under normoxic and hypoxic conditions using pimasertib, an allosteric inhibitor of MEK1 and MEK2 kinases [12].

**Figure 1:**
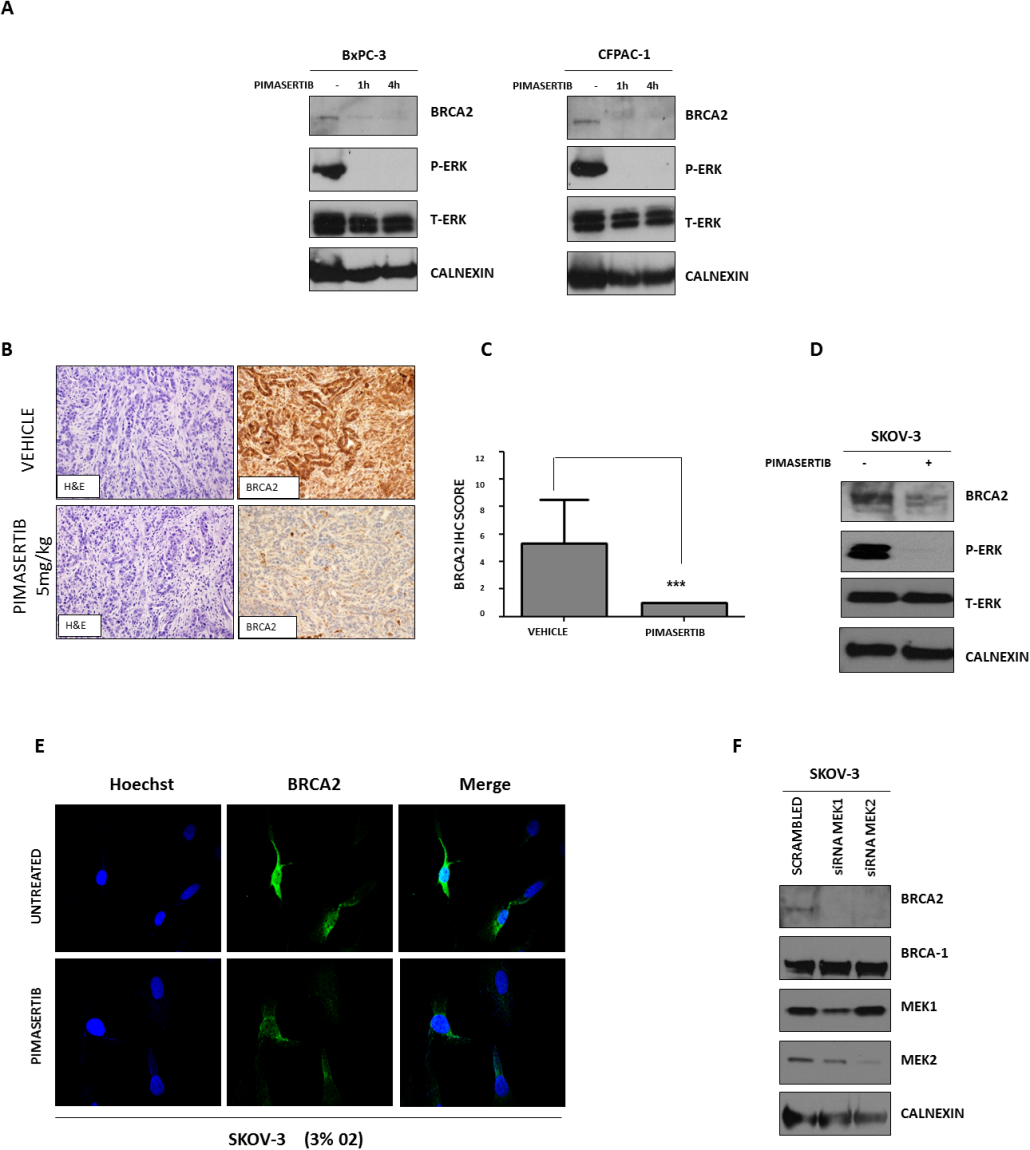
Effect of MEK inhibitor pimasertib on BRCA2 **(A)** Western blot analysis of P-ERK and homologous recombination repair protein BRCA2 expression in human pancreatic cancer cell lines after 1 and 4 hours of treatment with 0.5μM pimasertib under hypoxic conditions (3% O2). Data are representative of three independent experiments. **(B)** IHC staining images of BRCA2 on tumors derived from an orthotopic pancreatic model treated with 5 mg/kg pimasertib for 4 hours before sacrifice. Quantification of BRCA2 staining is shown in the column graph. **(C)** IHC scores were based upon the products of percentage positive cells multiplied by stain intensity (0 = negative, 1= weak, 2 = moderate, 3 = strong). **(D)** Western blot analysis of P-ERK and BRCA2 expression in human ovarian cancer cell lines SKOV-3 after 4 hours of treatment with 0.5μM pimasertib (3% O2). Data are representative of two independent experiments. **(E)** Immunofluorescence analysis of BRCA2 expression in human ovarian cancer cell line SKOV-3 upon 4h treatment with 0.5μM pimasertib under hypoxic conditions (3% O2). **(F)** Western blot analysis of BRCA2 and P-ERK signaling after transfecting SKOV-3 cell lines with 50 nM siRNA against MEK1 or MEK2 for 72 hours. Data are representative of two independent experiments.

Human pancreatic cancer cells lines (BxPC-3, CFPAC-1) were exposed to 0.5μM pimasertib under hypoxia (3% 02) for 4 hours and downregulation of BRCA2 protein expression was observed upon MEK inhibition (Figure 1A) whereas BRCA1 expression was unaffected (data not shown). We validated the effects of pimasertib on BRCA2 expression in an *in vivo* model by treating syngeneic pancreatic orthotopic xenografts derived from the KPC model of pancreatic cancer with pimasertib. Four hours after treatment, mice were sacrificed and tumors excised. Immunohistochemistry on these tumors confirmed downregulation of BRCA2 protein compared to vehicle-treated tumors (Figure 1B, 1C). Because reduction in BRCA expression represents a mechanism of sensitization to DNA damaging agents in ovarian tumors and has major therapeutic potential [13], we tested the activity of pimasertib in the BRCA-2 proficient human ovarian cancer cell lines SKOV-3. Western blot analysis showed that a 4-hour treatment with 0.5μM pimasertib resulted in down-regulation of BRCA2 concomitant with reduced ERK phosphorylation in these cells (Figure 1D). BRCA2 down-regulation upon pimasertib administration, under low oxygen conditions, was observed also by immunofluorescence (Figure 1E). The on-target effect of pimasertib was confirmed by using small interfering RNA (siRNA) targeting MEK1 and MEK2 proteins. As expected, siRNA-mediated depletion of MEK1 and MEK2 negatively regulated BRCA2 protein expression after a 72-hour transfection (Figure 1F).

### MEK inhibition impairs homologous recombination repair in BRCA2 proficient cells

**Figure 2:**
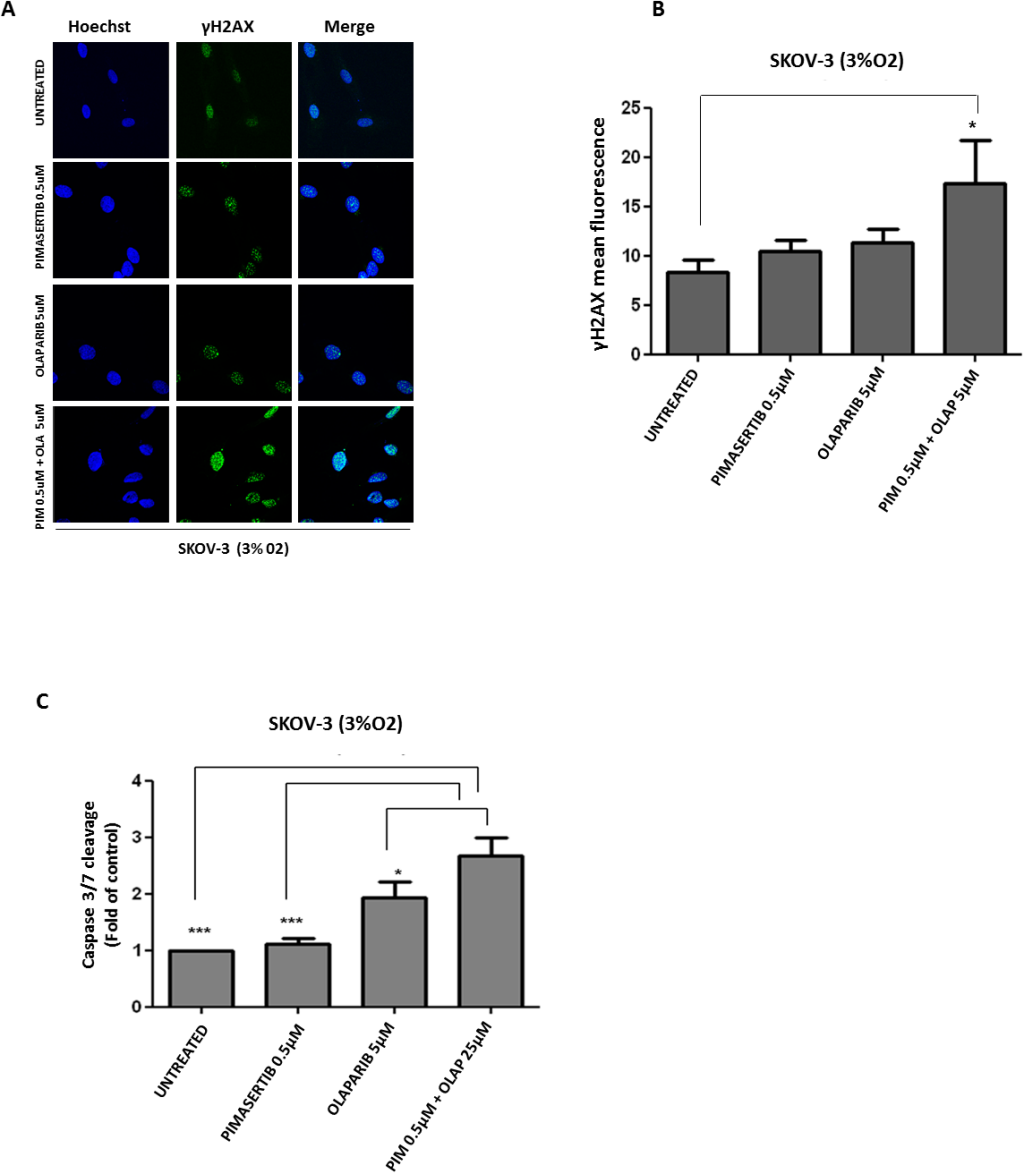
Enhanced DNA damage response and cytotoxicity after combination of PARP inhibitor olaparib with MEK inhibitor pimasertib in BRCA2 proficient cell line SKOV-3 **(A)** p-γ*H2AX* expression in SKOV-3 cells was detected by immunofluorescence after 24h treatment with 0.5μM pimasertib, 5μM olaparib or the combination of olaparib + pimasertib under hypoxic conditions. **(B)** Quantification of the yH2AX staining is shown in the column bar graph and expressed as mean fluorescence intensity. Data are shown as mean ± SD. Experiments were repeated three times. **(C)** Apoptosis upon 24h treatment with 0.5μM pimasertib, 25μM olaparib or their combination detected by measuring the levels of cleaved caspase-3 activity in SKOV-3 cell line. Data are means ± SD from three independent experiments. ^***^P<0.001, ^*^P<0.05 were considered statistically significant.

PARP inhibitors have been recently approved for the treatment of BRCA-deficient ovarian cancer patients [14]. Based on the findings showing suppression of BRCA2 expression upon MEK inhibition, we sought to investigate whether impaired HR caused by pimasertib in BRCA-proficient ovarian cancer cells would result in increased DNA damage following PARP inhibition. Immunofluorescence analysis of γH2AX nuclear staining, a read-out of DNA double strand breaks, was performed following 24 hours of drug treatment. Exposure with 0.5μM pimasertib plus 5μM olaparib (Figure 2A), enhanced γH2AX foci formation in the BRCA2 wild type SKOV3 cell line. Immunofluorescence signals were quantified as mean fluorescence intensity (Figure 2B). A similar effect on γH2AX foci formation was observed with the PARP inhibitor rucaparib (Supplementary Figure 1).

To evaluate whether the exacerbated DNA damage observed upon combined pimasertib and olaparib treatment translated to increased cell death, we measured Caspase 3-7 activity under these conditions. The addition of pimasertib enhanced olaparib-induced apoptosis by increasing the levels of caspase 3/7-enzyme activity compared to olaparib monotherapy in SKOV-3 cells under low oxygen concentrations (^*^P<0.05) (Figure 2C). Interestingly this occured only under hypoxic and not in normoxic conditions (ns P>0.05) (Supplementary Figure 2).

**Figure 3:**
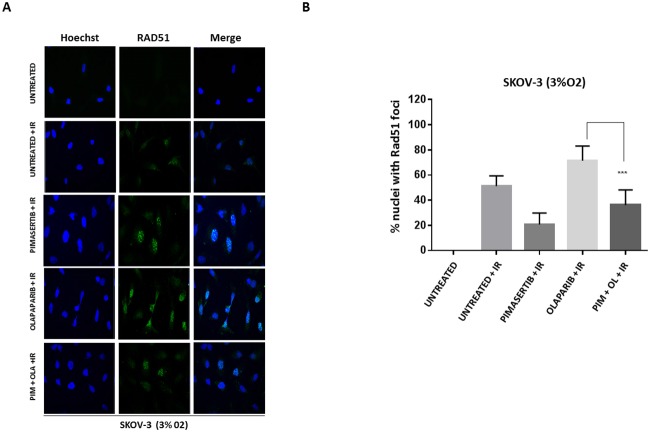
Pimasertib impairs RAD51 foci formation induced by olaparib in BRCA-2 proficient SKOV-3 cells **(A)** SKOV-3 cells were irradiated, treated for 4 hours with the indicated drugs and stained with anti-RAD51 ab. RAD51 nuclear foci after pimasertib, olaparib or the combination treatment were analysed by confocal microscopy in SKOV-3 cells. Representative images are shown. **(B)** the bar graphs represent the percentage of nuclei with RAD51 foci. Data are shown as mean ± SD. Experiments were repeated three times.

RAD51 is an essential component of HR. BRCA2 interacts with RAD51 and mediates its nuclear entry where it is then recruited to DNA break sites [15]. RAD51 forms foci whose formation is impaired in HR defective cells [16]. We observed increased formation of RAD51 nuclear staining in response to irradiation in BRCA2-proficient SKOV-3 cells, which further increased in the presence of olaparib (Figure 3A). Quantification of nuclei with RAD51 foci is shown (Figure 3B). The addition of pimasertib reduced formation of RAD51 foci in response to ionizing radiation alone and in combination with olaparib (Figure 3A), indicating that pimasertib impairs the repair of DNA double strand breaks by down-regulating BRCA2 under low oxygen conditions. MEK inhibition significantly reduced the formation of RAD51 foci in combination with olaparib in SKOV3 cells under hypoxic conditions (^***^P<0.001) (Figure 3A) but not under normoxia (Supplementary Figure 3).

**Figure 4:**
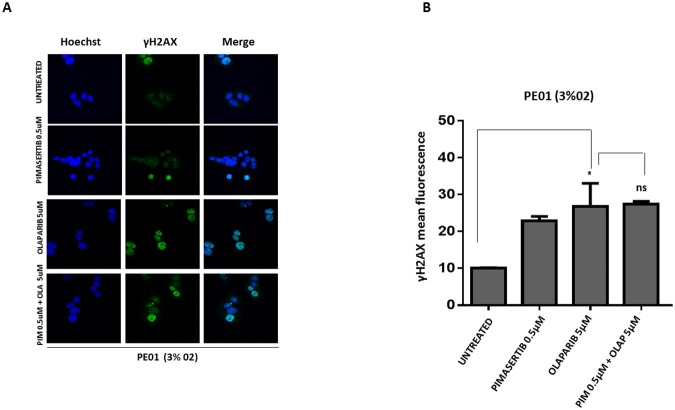
The cytotoxic activity of PARP inhibitors is not affected by the MEK inhibitor in BRCA2 mutant cell line PE01 **(A)** p-γ*H2AX* expression in PE01 cells was detected by immunofluorescence after 24h treatment with 0.5μM pimasertib, 5μM olaparib, the combination of olaparib + pimasertib under hypoxic conditions. Representative images are shown. **(B)** the bar graphs represent the quantification of p-γ*H2AX* foci. Data are shown as mean ± SD. Experiments were repeated three times.

We investigated whether the effects of MEK inhibition on downregulating BRCA2 and thus sensitizing to PARP inhibitor activity is restricted to cells with HR proficiency. Testing the effects of the combination in a BRCA2-mutant cell line (PE01) we found that olaparib monotherapy was sufficient to significantly increase the number of γH2AX foci compared to the control group, but addition of the MEK inhibitor did not enhance formation of γH2AX foci (Figure 4A and 4B). There was no increase in γH2AX protein levels upon 0.5μM pimasertib plus 5μM olaparib combination in PE01 cells (ns P>0.05) (Supplementary Figure 4). As expected, the combination of olaparib with pimasertib did not increase apoptosis compared to olaparib monotherapy in PE01 cells under low oxygen conditions as shown by the levels of cleaved caspase-3 activity (ns P>0.05) (Supplementary Figure 5).

### Pimasertib enhances the efficacy of both olaparib and evofosfamide

**Figure 5:**
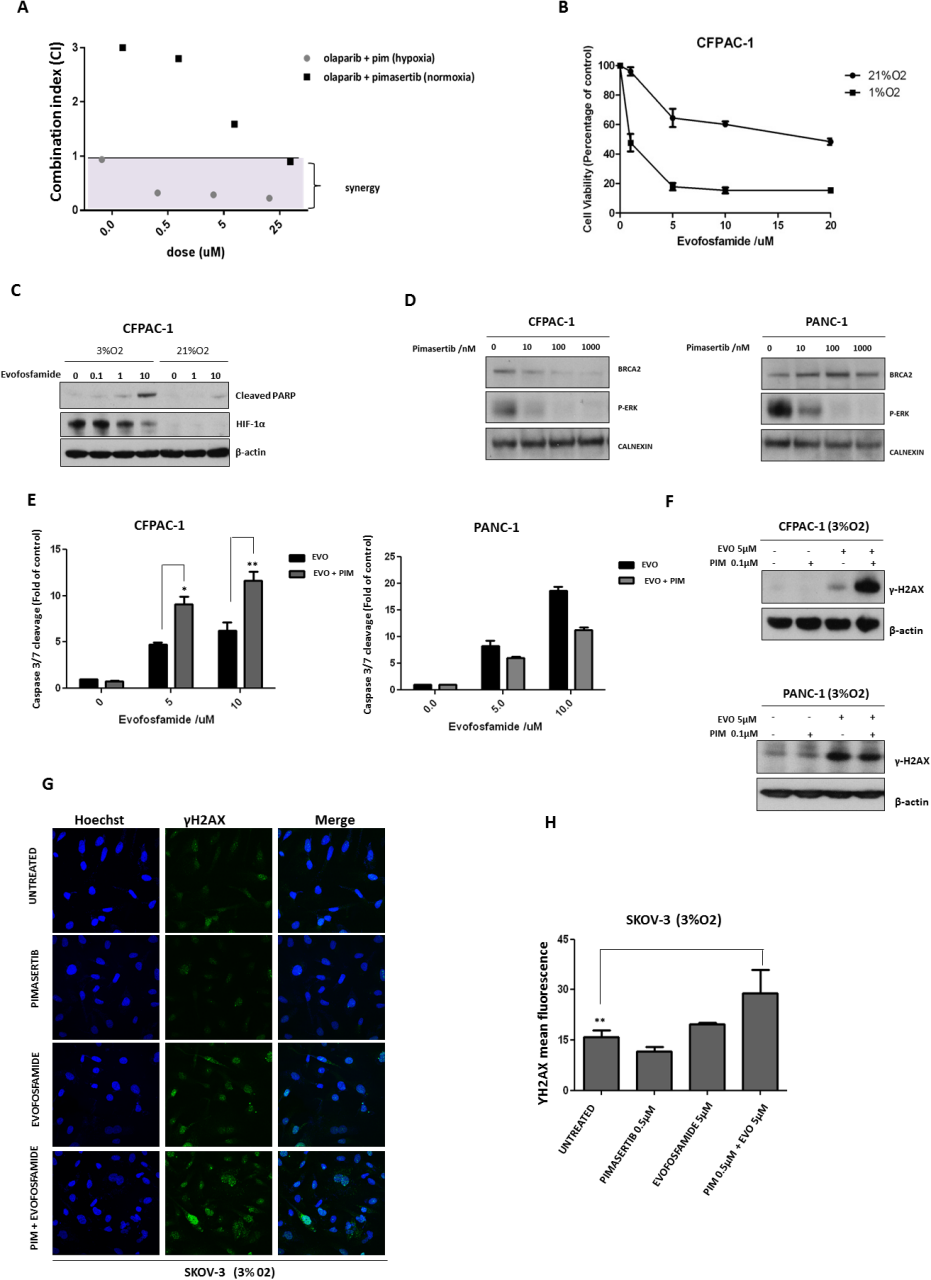
Pimasertib downregulates BRCA2 protein and sensitizes pancreatic cancer cells to the PARP inhibitor olaparib and the hypoxia-activated pro-drug evofosfamide **(A)** Cell viability after combination treatment of increasing concentrations of olaparib (5μM, 25μM, 50μM) with 0.5μM pimasertib in SKOV-3 cell lines was assessed by MTT assay. The calcusyn software was used to determine the combination index (CI) values at each concentration tested. CI>1 were considered antagonistic, CI<1 were considered synergistic. **(B)** Viability of CFPAC-1 cell lines treated with dimethylsulfoxide (DMSO) or increasing concentrations of evofosfamide for 72 hours (under hypoxia or normoxia), expressed as a percentage ± SD of three independent experiments. **(C)** Western blot analysis of cleaved-parp and HIF-1α expression after 24h treatment with increasing concentrations of evofosfamide under hypoxia or normoxia. **(D)** Western blot analysis showing the effects of pimasertib time course on BRCA2 and p-ERK expression in human pancreatic cancer CFPAC and PANC-1 cell lines. **(E)** Apoptosis upon 24h treatment with 0.5μM pimasertib, 5 or 10μM evofosfamide or their combination detected by measuring the levels of cleaved caspase-3 activity in CFPAC-1 and PANC-1 cell lines. Data are means ± SD from three independent experiments. ^**^P<0.01, ^*^P<0.05 were considered statistically significant. **(F)** Western blot analysis of yH2AX expression in CFPAC-1 and PANC-1 cell lines after 24 hours treatment with pimasertib, evofosfamide and their combination. Data are representative of three independent experiments. **(G)** p-γ*H2AX* expression in SKOV-3 cells was detected by immunofluorescence after 24h treatment with 0.5μM pimasertib, 5μM evofosfamide or their combination under hypoxic conditions. **(H)** the bar graphs represent the quantification of p-γ*H2AX* foci. Data are shown as mean ± SD. Experiments were repeated three times.

We next sought to confirm whether a synergistic interaction occurs between MEK and PARP inhibition. The effects of pimasertib in combination with olaparib on cellular proliferation were measured in SKOV-3 cells. Combination indices (CIs), were used to determine the synergism potential and were calculated using the methodology of Chou and Talalay [17] with the Calcusyn Software. A fixed dose of 0.5μM pimasertib with increasing concentrations of olaparib (0.5μM, 5μM, 25μM) produced synergistic antiproliferative activity in SKOV-3 cells under hypoxia (CI values <1), but not under normoxia as shown by the CI values being almost all higher than 1 (Figure 5A). These results suggest that MEK inhibition could represent a strategy to enhance PARP inhibitor activity in BRCA2 proficient tumors by decreasing BRCA2 expression.

Given that tumors are characterized by hypoxic regions that determine drug resistance and accelerate cancer cell proliferation [18], the use of hypoxia-activated prodrugs represents a potentially important strategy to target the hypoxic tumor microenvironment. Evofosfamide, a nitroimidazole-linked prodrug of a brominated version of isophosphoramide mustard (Br-IPM) that acts as a DNA cross-linking agent, is selectively activated under hypoxia and has shown antitumor activity [19, 20]. We investigated whether MEK inhibition would potentiate the activity of the hypoxia-activated drug evofosfamide by increasing DNA damage with the combination. First, the effects of evofosfamide on the human pancreatic CFPAC-1 cell line viability under low or high oxygen concentrations were compared. As expected, evofosfamide decreased cell viability of these cells under hypoxic conditions (Figure 5B). Western blot analysis showed increased cleavage of PARP, following 24h treatment with evofosfamide (0.1μ, 1μM, 10μM) under hypoxia (Figure 5C). Similar to BxPC-3 and SKOV-3 cells, pimasertib treatment reduced BRCA2 expression in CFPAC-1 cells (Figure 5D) resulting in increased apoptosis upon combination of pimasertib with olaparib compared to single agents alone (Figure 5E). Interestingly, this effect was not observed in PANC-1 cells, in which BRCA2 did not decrease following pimasertib treatment (Figure 5D, 5E). Accordingly, western blot analysis increased expression of phosphorylated H2AX upon pimasertib plus evofosfamide treatment in CFPAC-1 cells but not in PANC-1 cells (Figure 5F), suggesting that enhanced cytotoxicity of the drug combination is dependent on the effect of MEK inhibition through inhibiting BRCA2.

We also tested the effect of combining the MEK inhibitor pimasertib with evofosfamide in the ovarian cancer cell lines SKOV-3. Our results demonstrated that pimasertib with evofosfamide promoted DNA damage in SKOV-3 cells compared to the single agents alone as shown by the accumulation of phosphorylated H2AX nuclear foci formation (Figure 5G). Quantification of γH2AX nuclear foci showed significant increase in the combination-treated cells as compared with vehicle-treated cells (Figure 5H, ^**^P<0.01).

### Pimasertib enhances the efficacy of evofosfamide *in vivo* in BRCA wild type but not BRCA aberrant xenograft models

We next sought to confirm whether a synergistic interaction occurs between pimasertib and evofosfamide in an *in vivo* setting. *In vivo* models were selected to also evaluate the impact of BRCA status on the combination activity of pimasertib and evofosfamide. The MiaPaCa-2 cell line has no known genetic defects in BRCA2. BxPC3 has been described to harbour a loss of heterozygosity for the BRCA2 locus. Three different sequences of administration were tested for the pimasertib plus evofosfamide combination: pimasertib 30 mg/kg PO (per os) QD and evofosfamide 75 mg/kg IP (intraperitoneally) Q3D dosed simultaneously; pimasertib 30 mg/kg PO QD dosed 2 hours before evofosfamide 75 mg/kg IP Q3D; evofosfamide 75 mg/kg IP Q3D dosed 2 hours before pimasertib 30 mg/kg PO QD.

**Figure 6:**
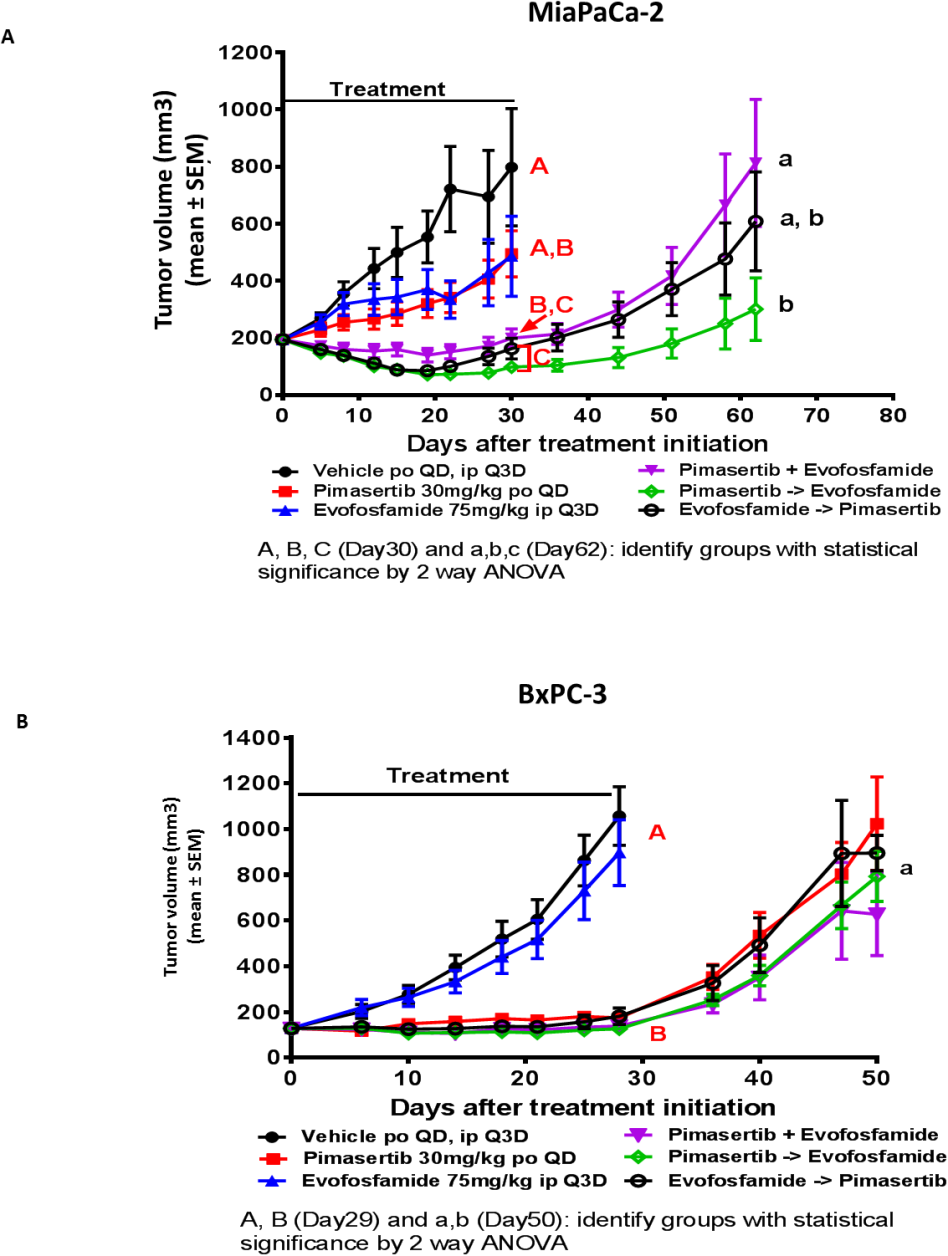
The combination of evofosfamide with pimasertib increases the anti-tumor response beyond the activity of each single agent alone in BRCA wt but not BRCA aberrant xenograft models The antitumor activity of pimasertib and evofosfamide were evaluated as single agents and in combination. Three combination sequencing schedules were tested: simultaneous administration, pimasertib 1 hour prior to evofosfamide administration, and evofosfamide 1 hour prior to pimsertib administration. Tumor regrowth phase was performed in treatment groups where tumor stasis or regression were observed at end of treatment. **(A)** MiaPaca-2 xenograft model. **(B)** BxPC3 xenograft model. (P<0.05 were considered statistically significant).

In the MIAPaCa-2 xenograft model, there was no significant tumor growth inhibition in either the pimasertib or evofosfamide monotherapy groups (ns p>0.05). Tumor growth inhibition was observed in all three combination groups, but there was no statistically significant difference among them at the end of the treatment (Day 30). On Day 30, the vehicle and single agent groups were terminated and the combination groups were allowed to continue for analysis of tumor re-growth kinetics. At the end of the study, on Day 62, the combination sequence of pimasertib given first and followed by evofosfamide showed a reduction in tumor volume compared to the sequence of pimasertib and evofosfamide given simultaneously (p<0.05) (Figure 6A). All treatments were well tolerated.

In the BxPC3 xenograft model, evofosfamide as a single agent did not have a significant effect on tumor growth, but pimasertib alone and the combination groups all significantly inhibited tumor growth. On Day 29 after measurements were recorded, the vehicle and evofosfamide groups were terminated and the pimasertib-treated as well as the combination groups were allowed to continue for a tumor growth delay phase. When the study was terminated on Day 50, tumors in all groups were growing and there was no significant difference between the pimasertib monotherapy and the combination groups. In addition, there was no significant difference in efficacy among the 3 combination groups with the 3 different sequences of administration (simultaneous, pimasertib first or evofosfamide first) (Figure 6B). All treatments were well tolerated.

## DISCUSSION

Mutation of components of the Ras/Raf/MEK/ERK signalling pathway frequently occurs in human cancer and targeting this cascade with small-molecule inhibitors is an established therapeutic strategy. The antitumor activity of MEK inhibitors have been assessed in several preclinical and clinical studies and holds promise as cancer therapeutics [21, 22], with some achieving clinical success [23]. MEK inhibitors have been developed clinically and have shown efficacy in several cancers including melanomas [23]. However, the overall efficacy of monotherapy with MEK inhibition in treatment of human cancers has been limited. There has therefore been interest in combination strategies which improve the effectiveness of this approach. A recent study demonstrated that KRAS and NRAS, and some BRAF, mutant tumors are sensitive to MEK inhibition in combination with PARP inhibition [24].

Previously, it was reported that hyperactivation of the RAS/MEK/ERK pathway contributes to radioresistance in tumors by enhancing the repair of double strand DNA breaks induced by radiotherapy [25]. Targeting MEK has been shown to impair DNA damage repair pathways and sensitize tumor cells to radio and chemotherapy. For instance, MEK inhibitors have been demonstrated to interfere with both HR and NHEJ pathways, thus rendering pancreatic cancer cells more sensitive to ionizing radiation [26].

PARP inhibitors are an approved treatment in patients with mutations of the BRCA1/2 genes. However, only a small fraction of all cancer patients carry a BRCA1 or BRCA-2 deficiency, making PARP inhibitors efficacious in only a limited subset of patients [27].

In this study we demonstrated that combining the MEK inhibitor pimasertib with the PARP inhibitors olaparib and rucaparib increased DNA damage and induced antiproliferative responses in BRCA2-wild type ovarian cancer cell lines, indicating that MEK inhibitors could be used as a therapeutic strategy to produce a ‘BRCAness’ phenotype [9] that would sensitize BRCA2 wild-type proficient tumors to PARP inhibition.

The effectiveness of combination treatment combining MEK and PARP inhibition is consistent with a study of Mills et al showing that K-RAS and N-RAS mutant tumors are exquisitely sensitive to the combination of PARP and MEK inhibitors. In that study, there was increased cell death caused by MEK inhibitor-induced FOXO3 activation, modulated by a BIM-mediated apoptotic response [24].

RAD51 is a protein essential for HR repair in mammalian cells [28]. BRCA2 directly interacts with RAD51 upon DNA damage and mediates its translocation into the nucleus at the site of damage [15]. Patients carrying a BRCA mutation benefit from PARP inhibitors [8]. An increase in the number and size of RAD51 foci is associated with genomic damage. In this study, we showed that MEK inhibition impaired HR repair, reduced the formation of a marker of HR, RAD51, thus enhancing DNA damage after olaparib treatment.

The ability of pimasertib to impair HR through the down-regulation of BRCA2 is a determinant factor of the efficacy we observed between PARP and MEK inhibitors and with evofosfamide. Although the effect on the modulation of BRCA2 likely occurs through a posttranslational modification, as we observed rapid down-regulation of its protein levels after MEK inhibition, understanding the precise mechanism underlying the inhibition of BRCA2 expression will be crucial and is currently under investigation.

The hypoxic microenvironment found in solid tumors such as ovarian and pancreatic cancer, influences response to treatment [29]. Hypoxic tumor cells are associated with increased resistance to radiotherapy and chemotherapy [30]. Previous studies have reported that PARP inhibitors exert greater cytotoxicity in hypoxic cells by suppressing the expression of DNA repair molecules [31]. In addition, in NSCLC models, a hypoxic environment was also shown to result in radiation-sensitizing effects of PARP inhibitors [32]. In this study, we demonstrated the impact of hypoxia on the effects caused by the MEK inhibitor pimasertib and found synergistic antitumor activity in combination with PARP inhibition specifically under hypoxic conditions.

Because of this selective activity of MEK and PARP inhibitors in hypoxia, we further investigated the combination effects of pimasertib with the hypoxia-activated agent evofosfamide [33]. There was significant increased apoptosis in pancreatic cancer cell lines in which the MEK inhibitor caused inhibition of BRCA2 expression and enhanced antitumor activity in BRCA2 wild-type pancreatic xenograft tumors treated with evofosfamide plus pimasertib, compared with cancers treated with evofosfamide alone.

These results suggest that hypoxia-activated drugs could be combined synergistically with MEK inhibition. Our findings indicate that combining DNA damaging agents with MEK inhibition could represent a potential strategy to target HR-proficient tumors, especially in the presence of hypoxia.

## MATERIALS AND METHODS

### Reagents

The MEK1/2 inhibitor pimasertib and hypoxia-activated prodrug evofosfamide were provided by EMD Serono Research and Development Institute (Billerica, MA) and Threshold Pharmaceuticals (South San Francisco, CA). Both reagents were dissolved in DMSO to make a 10mM stock solution and were stored at −20°C. The PARP inhibitors olaparib and rucaparib were a gift from the University College Hospital MacMillan Cancer Center (London, UK). The following reagents were used: Thiazolyl Blue Tetrazolium Bromide (MTT); for immunoblotting, the following antibodies were used: anti–β-actin and anti-calnexin as loading controls; anti-cleaved-PARP, anti-p-ERK, anti-ERK, anti-γH2AX (Cell Signaling Technology) and secondary antibodies anti-mouse and anti-rabbit IgG HRP linked antibodies (Cell Signaling Technology). For Immunofluorescence analysis, anti-BRCA2 (Santa Cruz Biotechnology), anti-RAD51 (Abcam) and anti-phosphorylated histone-H2AX (Merck Millipore) antibodies were used.

### Cell line and culture conditions

PANC-1, BxPC-3, MiaPaca-2, CFPAC-1 human pancreatic cancer cells and SKOV-3, MiaPaCa-2, PE01 human ovarian cancer cell lines were purchased from the American Type Culture Collection (ATCC; Manassas, VA, USA). PANC-1, SKOV-3, MiaPACA-2 and CFPAC-1 cell lines were grown in Dulbecco’s minimal essential medium (Autogen Bioclear), PE01 and BxPC-3 cell lines were grown in RPMI-1640 medium (Autogen BioclearAll cells were supplemented with 10% fetal bovine serum, 5% glutamine and 5% Penicillin/Streptomycin and incubated at 37°C in 5% CO_2_. Cells used in the *in vivo* studies were cultured in the absence of antibiotics.

### Immunoblotting

Protein extracts were prepared with the CelLytic™ M cell lysis reagent (Sigma-Aldrich). 35μg of protein were denatured by heating for 5 min at 95μ C in sample buffer containing 100 mM Tris-Cl (pH 6.8), 4% SDS, 10% 2-mercaptoethanol, 20% glycerol, and 0.02% bromophenol blue (Life Technologies) and resolved on a 4-12% Bis-Tris NuPAGE gel (Life Technologies). Proteins were subsequently transferred to polyvinylidene difluoride membranes (Immobilon®-P transfer membrane; Millipore) in 1X-Tris-Glycine-20% Methanol transfer buffer. Membranes were blocked for 1h at room temperature in blocking buffer containing 5% BSA (Sigma-Aldrich) in 1x TBS, 0.1% Tween-20. All primary antibodies were incubated overnight at 4°C. Anti-rabbit or mouse IgG, HRP-linked Antibody (Cell Signaling Technologies) were used to detect primary antibody binding. The binding was visualized by ECL chemiluminescent detection reagent (Amersham) on autoradiography film (Kodak-X-Omat).

### Apoptosis assays

Apoptosis was measured by assessing Caspase 3/7 enzyme activity with the Caspase 3/7 Glo assay (Promega) according to the manufacturer’s protocol. Luminescence was measured with the Varioskan Flash Multimode Reader (Thermo Scientific) and values were normalized to untreated control and presented as fold increase of control.

### Drug combination analysis

4,000 cells/well were seeded in a clear, flat bottom 96 well plate (Corning). The following day, cells were treated with olaparib (0.05μM, 5μM, 25μM, 50μM) or pimasertib (0.01μM, 0.5μM, 1μM, 10μM) for 72 hours or with a fixed dose of pimasertib (0.5μM) plus increasing concentrations of olaparib for 72 hours, before harvesting. All drugs were diluted in cell culture media. Following drug treatments, cells were incubated with 20μL/well of MTT (3-[4, 5-dimethylthiazol-2-yl]-2, 5 diphenyl tetrazolium bromide) (5 mg/mL) for 4h at 37°C to detect cell proliferation. Formazan crystals were solubilised in 200μL of DMSO and the absorbance was measured at 540 nM with the Varioskan Plate reader.

The interaction between olaparib and pimasertib was evaluated by using The Calcusyn Software according to the Chou and Talalay method [17]. This analysis produces a combination index value (CI), with values less than 1 indicating synergism, greater than 1 indicating antagonism.

### Immunofluorescence

Cells were seeded on glass coverslips placed on 24-well plates. 24 hours after plating, the cells were treated with pimasertib, olaparib or the combination for 4 or 24 hours. After treatment, cells were fixed in paraformaldehyde for 10 minutes, washed twice, permeabilized with 0.1% Triton X-100 for 5 minutes, and blocked in PBS with 5% BSA (Sigma) for another 60 minutes. The γH2AX antibody was added for 1 hour at room temperature, followed by 3 washes in PBS and 30 minutes incubation with ALEXA Fluor 488 secondary antibody. After 3 washes of 5 minutes each, HOECHST (Thermo Fisher Scientific) at 1 mg/ml in PBS was used to stain nuclei, applying it for 30 minutes. Finally, glass coverslips were mounted using fluoromont (Sigma Aldrich) and observed under a Leica microscope using Leica software. γH2AX fluorescence was quantified using Imaris software, where at least 50 nuclei were analysed. A cell with more than 5 nuclear foci was considered positive. The same criterion was applied for the analysis of RAD51 foci. RAD51 count was the number of RAD51 positive cells divided by the total number of cells expressed as percentage.

### Immunohistochemical (IHC) staining

For immunohistochemical analysis, 4mm paraffin sections underwent automated dewaxing (Leica Bond Dewax AR9222) and endogenous peroxidase was blocked using 3-4% (v/v) hydrogen peroxide (part of Leica Bond Refine Polymer Kit, DS9800). Automated antigen retrieval was then performed on the sections. For BRCA2 Leica Bond ER2 (EDTA-based, pH9, AR9640) was applied to the slides and they were heated to 100 degrees Celsius (30 minutes). The antibody was used on the slides obtained from mice pancreatic tumors at a dilution of 1/100 with 15 minutes incubation. Signal visualization was performed using Bond Polymer (Anti-rabbit Poly-HRP-IgG) for 8 minutes. DAB was applied for 10 minutes and then Bond DAB Enhancer (Copper Sulfate-based, AR9432) was applied for 5 minutes. Cell nuclei were counterstained with haematoxylin. The Leica Bond Polymer Detection Kit (DS9800) was used for peroxidase blocking, visualization and counterstaining. Bond Wash (AR9590) was used for all washing steps between reagent steps.

### IHC quantification

Immunostaining for BRCA2 was assessed in at least five fields at 400× magnification. Immunoreactivity was evaluated semi-quantitatively based on staining intensity and proportion. The proportion of staining was scored from 0 to 3 as follows: 3: >50% of cells positive; 2:10-49%; 1: <10%. Intensity of staining was scored from 0 to 3 (0, absent; 1, weak; 2, moderate; 3, intense). The immunoreactive score for each sample was determined by multiplying the intensity and the proportion of stained cells. Analysis was undertaken blindly without knowledge of treatment variables.

### Statistical analysis

One-way ANOVA, Two-way ANOVA and Bonferroni post-tests were used to calculate statistical significance for the *in vitro* experiments. Student’s *t*-test was used to calculate statistical significance of tumor weight. ^*^P <0.05, as calculated by GraphPad Prism (version 6.0; GraphPad Software Inc.), were considered statistically significant.

### *In vivo* xenograft study

MiaPaCa2 cells (10×10^6^ in a 200μl PBS:Matrigel (1:1) suspension) were subcutaneously injected into the right shoulder area of female nude (Crl:NU-Foxn1nu) mice (6-8 weeks old, Charles River Laboratories, Wilmington, MA). BxPC3 cells (5×10^6^ in a 200μl suspension) were subcutaneously injected into the right shoulder area of female NIH III nude (Crl: NIH-*Lyst ^bg^Foxn1^nu^Btk ^xid^*) mice (6-8 weeks old, Charles River Laboratories, Wilmington, MA). Mice were housed and maintained in individually-ventilated cages under specific pathogen-free conditions and received a standard diet with free access to water. All mice were acclimated for at least 48 hours prior to study initiation and were used according to the guidelines approved by the EMD-Serono Institutional Care and Animal Use Committee (IACUC), #11-011.3.2.3.

### Evaluation and statistical analysis of *in vivo* studies

Efficacy was determined by analysing tumor volumes and percentage of ΔT/ΔC (% ΔT/ΔC). Tumor volume was determined by using the tumor length (l) and width (w) measurements and calculating the volume with the equation l^*^w2/2. The length was measured along the longest axis of the tumor and width was measured perpendicular to that length. The mean percent of %ΔT/ΔC was calculated as follows: [%ΔT/ΔC= ((TVf - TVi)/(TVfCtrl – TViCtrl)) x 100%], where TV=tumor volume, f=final, i=initial and Ctrl=control group. In the instances where tumors regressed, %ΔT/ΔC was calculated as follows: [%ΔT/ΔC= ((TVf - TVi)/TVi) x 100%], where TV=tumor volume, f=final, i=initial for that particular group.

Tumor volume data were analyzed by Repeated Measure Two-Way Analysis of Variance (RM-ANOVA) followed by Tukey’s Bonferroni post-hoc multiple pair-wise comparisons (α = 0.05) using Prism 5.02 software from GraphPad. Tumor volume data used for statistical analyses were log-transformed before performing the RM-ANOVA and Day 0 data were left out of the analysis because there was no effect due to treatment on this day. For the treatment phase of the study statistics were performed using the data up to Day 30 and for the re-growth phase (the three combination groups), statistics were performed using the data up to Day 62 before mice were euthanized due to the endpoint.

## SUPPLEMENTARY MATERIALS FIGURES AND TABLES


