# Supplementary material for: MEK inhibition leads to BRCA2 downregulation and sensitization to DNA damaging agents in pancreas and ovarian cancer models
Source: Oncotarget. 2018 Jan 22;9(14):11592–603. doi: 10.18632/oncotarget.24294 (PMC5837749; doi:10.18632/oncotarget.24294)
Supplement: Supplementary file 1 [file oncotarget-09-11592-s001.pdf]

MEK inhibition leads to BRCA2 downregulation and sensitization to DNA damaging agents in pancreas and ovarian cancer models

SUPPLEMENTARY MATERIALS

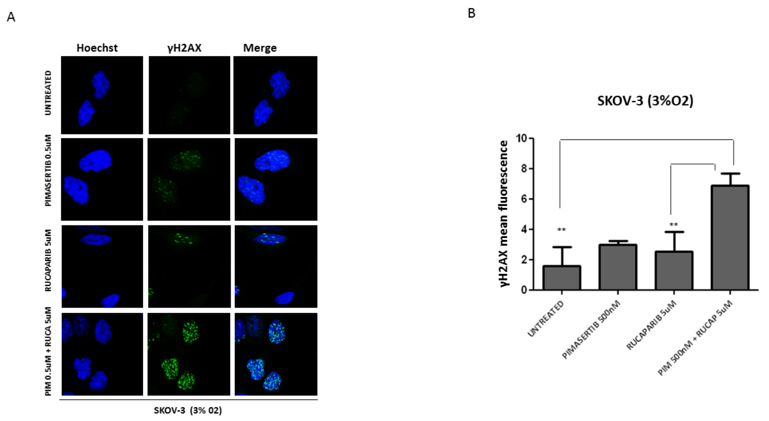

Supplementry Figure 1: Enhanced DNA damage response after combination of rucaparib with pimasertib in BRCA2 proficient cell line SKOV-3. (A) γ*H2AX* expression in SKOV-3 cells was detected by immunofluorescence after 24h treatment with 0.5µM pimasertib, 5µM rucaparib or the combination of rucaparib + pimasertib under hypoxic conditions. **(B)** Quantification of the γH2AX staining is shown in the column bar graph and expressed as mean fluorescence intensity. Data are shown as mean ± SD. Experiments were repeated three times.

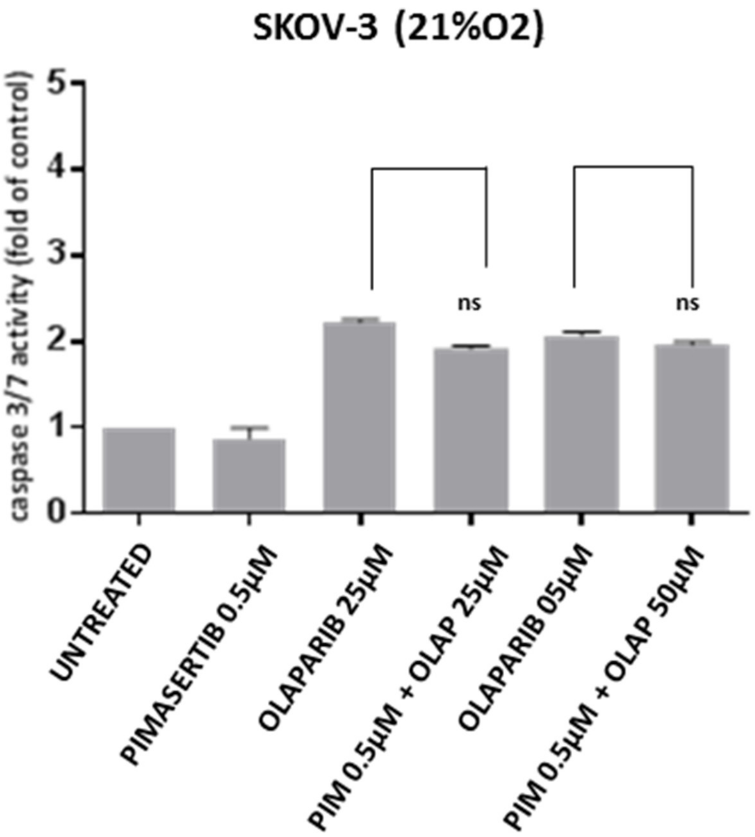

Supplementry Figure 2: Effects of pimasertib in combination with olaparib on apoptosis under normoxia. Apoptosis upon 24h treatment with 0.5µM pimasertib, 25-50µM olaparib or their combination detected by measuring the levels of cleaved caspase-3 activity in SKOV-3 cell lines. Data are means ± SD from three independent experiments. P>0.05 was considered non-statistically significant.

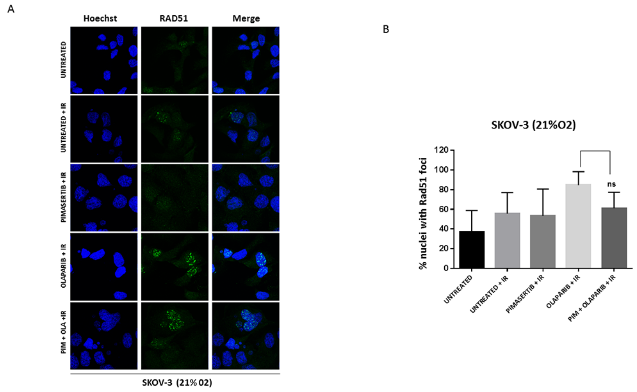

Supplementry Figure 3: Pimasertib does not affect RAD51 foci formation in response to olaparib under normal oxygen conditions. **(A)** Cells were irradiated, treated for 4 h with the indicated drugs at 21% O_2 and stained with anti-RAD51 ab. RAD51 nuclear foci after pimasertib, olaparib or the combination treatment were analysed by confocal microscopy in SKOV-3 cells. Representative images are shown. **(B)** The bar graph represents the percentage of cells with RAD51 foci. Data are shown as mean ± SD. Experiments were repeated three times.

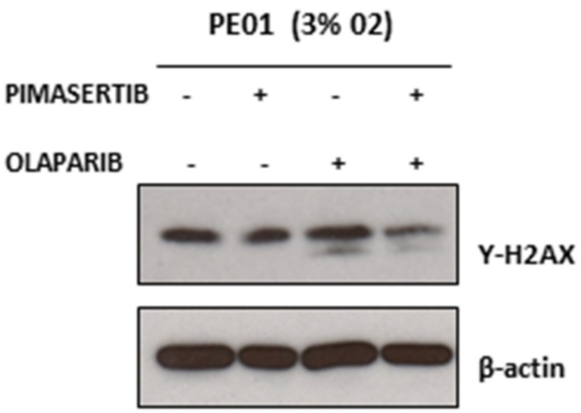

Supplementry Figure 4: Effect of pimasertib plus olaparib combination on γH2AX expression in BRCA2-deficient cell line PEO1. γH2AX protein expression in PE01 cells was detected by immunoblotting after 24h treatment with 0.5µM pimasertib, 5µM olaparib or the combination.

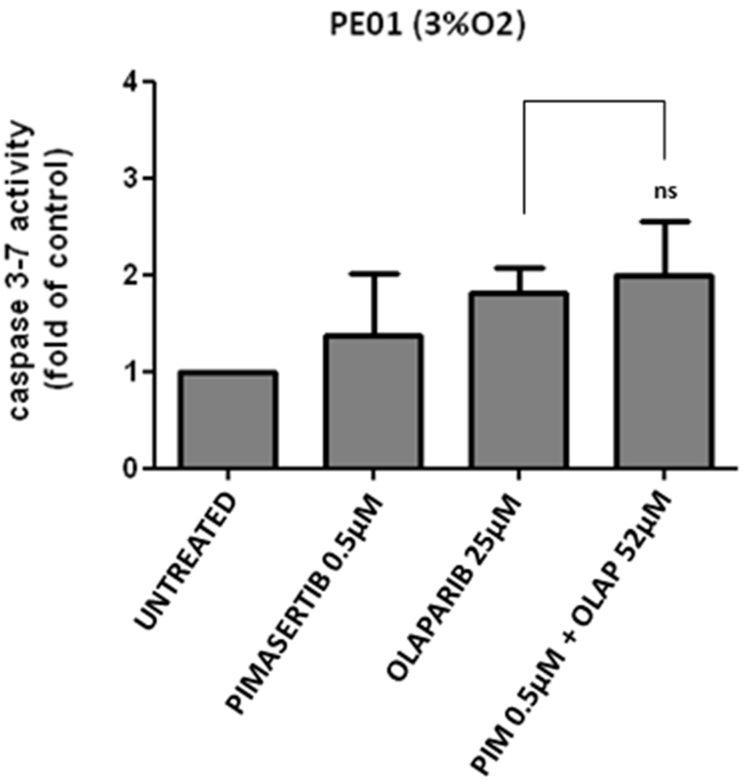

Supplementry Figure 5: Apoptosis by olaparib with pimasertib in PEO1 cells. Apoptosis upon 24h treatment with 0.5µM pimasertib, 25µM olaparib or their combination detected by measuring the levels of cleaved caspase-3 activity in PE01 cell line. Data are means ± SD from three independent experiments. P>0.05 was considered non-statistically significant.
